# Childhood epilepsy: Management in resource-limited setting

**DOI:** 10.4103/0972-2327.40223

**Published:** 2008

**Authors:** Chhaya Valvi, Subhashchandra Daga, Ujwala Kabade, Madhuri Agarwal

**Affiliations:** Department of Pediatrics, B.J Medical College and Sassoon General Hospital, Pune, India

**Keywords:** Childhood epilepsy, phenobarbitone

## Abstract

**Objective:**

To optimize the use of phenobarbital and/or phenytoin as frontline drugs for treatment of childhood epilepsy.

**Design:**

Before-and -after study.

**Setting:**

Epilepsy clinic at paediatric OPD, Sassoon General Hospital, Pune.

**Materials and Methods:**

Epilepsy is a condition in which seizures are triggered recurrently from within the brain. For epidemiological classification purpose epilepsy is considered to be present when two or more unprovoked seizures occur at an interval greater than twenty four hours apart. Seizures were classified as generalized and partial seizures, with underlying etiology investigated with EEG, CT scan in majority of the patients. Follow - up rate, seizure - control and antiepileptic drugs used among 151 children enrolled as on 31 March 2005 were compared with 106 children with new onset epilepsy enrolled as on February 2006. Eight children with breakthrough convulsion after a seizure free period of five to eighteen months were followed up after injection vitamin D. Nineteen children with poor control of seizures receiving polytherapy with newer antiepileptic drugs were assessed with frontline antiepileptic medication of phenobarbital and/or phenytoin. Serum calcium, phosphorus, alkaline phosphatase were done in seventy two consecutive children with seizure disorder.

**Results:**

During post protocol period good seizure control was achieved in 84.8% as against 80.7% and use of phenobarbital and/or phenytoin increased to 65.11% from 22.87%. Of the 8 cases with breakthrough seizures seven remained seizure free after vitamin D administration and with no dose enhancement of AED medications of the nineteen. Children receiving polytherapy thirteen children could be successfully switched to phenobarbital and/or phenytoin. Forty four (61%) children had hypocalcemia (less than 9 mg%), fifty seven (79%) children had raised alkaline phosphatase levels (more than 270 IU).

**Comments:**

Phenobarbital and/or phenytoin have been found to be effective frontline AED. Periodic administration of vitamin D plays a supportive role.

## Introduction

Epilepsy is the most common neurological problem in children. This disorder can be successfully controlled with a single well-tolerated anti-epileptic drug (AED) in most cases. It is estimated that 80 percent of the epilepsy cases are in developing countries. The treatment gap i.e. the percentage of those with epilepsy who are receiving no or inadequate treatment, is 90% in developing countries,[1] 70% in rural India[2] and 57% in South African children.[3] Choice of right AED and its easy availability can substantially reduce the treatment gap. Phenobarbital has become WHO's frontline AED for partial and generalized tonic - clonic seizures in developing countries[4] by virtue of its cost, efficacy and suitability.[5] In fact, studies in developed countries have shown that, as far as efficacy is concerned, there is little to choose between phenobarbital, phenytoin, carbamazepine, primidone or valproic acid in partial seizures with or without secondary generalization.[6,7] Keeping these facts in mind we studied the feasibility of making phenobarbital and/or phenytoin as frontline option at our epilepsy clinic.

## Materials and Methods

One hundred and fifty one children who were registered at and were attending our epilepsy clinic during the pre-protocol period with 106 children with new-onset epilepsy registered prospectively between February 2006 and January 2007. We used the following working definition of epilepsy. Epilepsy is a condition in which seizures are triggered recurrently from within the brain. For epidemiological classification purpose epilepsy is considered to be present when two or more unprovoked seizures occur at an interval greater than twenty four hours apart.[8] Seizures were classified as generalized and partial seizures. ILEA classification was not applied to all children. Underlying etiology was investigated with EEG, CT scan in majority of the children in the pre-protocol period. In post protocol period EEG was done in all children, CT scan was done in selective cases. Type of seizures, follow - up rate, seizure - control and antiepileptic drugs recieved among children enrolled were recorded.

During intervention phase first phenobarbital replaced other AED in children without neurodevelopmental handicap and in those children with poor control of convulsions. Phenytoin was added when convulsions were partially controlled with phenobarbital at 6mg/kg/day after a trial period of 4 weeks which was the follow up visit for all children. A close watch was kept on behavioral change and excessive sedation after starting phenobarbital. Phenobarbital was replaced when behavioral change was unacceptable like excessive sedation and hyperactivity. Mother's or caregiver's assessment was given maximum weightage, no formal neuropsychological assessment was done. If hyperactivity was coming forth as a complaint it had received the threshold for change. Those children with a risk of drop out were changed to phenobarbital and/or phenytoin. Long distance stay, irregular in treatment, ageing grandparents coming to collect medicines, went into perception of this risk. The main consideration was good control with avoidance of risk of drop out. New-onset cases received phenobarbital and/or phenytoin. Nineteen referred cases were for poor seizure-control despite polytherapy that included carbamazepine, clobazam, lamotrigine and valproate in varying combinations. An attempt was made to switch them to phenobarbital and/or phenytoin.

In seventy two children with seizures serum calcium, phosphorus, alkaline phosphatase was estimated. Vitamin D was administered parenterally every three months, 6,00,000 IU to children above five years and 3,00,000 IU to the younger ones. Before changing the AED, Vitamin D was given and children observed till the next follow-up visit a month later.

## Results

During pre-protocol phase, seizures were generalized in 77.1% and partial in 22.9%, children. Eighty-three (55.3%) children were following up and sixty-seven (44.6%) had dropped out. Eighty children (96.38%) had good follow up (9 and more visits/12 expected visits), three (3.6%) had poor follow up (less than 9 visits). Seventy nine children (95.18%) had achieved good control (no convulsions)/or fair control (one convulsion every month or two), one (1.2%) had poor control (frequent convulsions). Fourteen children (16.81%) were receiving phenobarbital, three (3.61%) phenytoin, forty three (51.8%) carbamazepine and others twenty one (25.3%) were receiving other AED.

During post-protocol period, one hundred and six children with new-onset epilepsy received treatment as per above protocol. Eighty six (81.1%) subjects followed up and twenty (18.8%) dropped out. Of these, eighty four patients (97.67%) had good follow up and two (2.3%) have poor follow up [see Table 1]. Eighty three (96.8%) children had good control of seizures and one(1.16%) has poor control. Forty seven children (54.65%) were receiving phenobarbital, four (4.65%) phenytoin, five (5.81%), phenobarbital and phenytoin. Eighteen (20.93%) were receiving carbamazepine and twelve(13.95%) were receiving other AED. Follow up of children had appreciably improved. Follow up rate is an imprecise but a good indicator of efficacy. Thus, in ninety five percent cases convulsions could be controlled with an easy-to-follow protocol.

**Table 1:** Comparison of pre-protocol (March 2005) and post-protocol (January 2007) parameters

	Pre-protocol	Post-protocol
Number of patients enrolled	150	106
Dropped out	67 (44.6)	20 (18.8)
Followed up	83 (55.3)	86 (81.1)
Follow up - Good	80 (96.3)	84 (97.6)
Poor	3 (3.6)	2 (1.8)
Control of seizures		
Good	67 (80.7)	73 (84.8)
Fair	12 (14.4)	10 (11.6)
Poor	1 (1.2)	1 (1.16)
AED*		
Phenobarbital	14 (16.86)	47 (54.65)
Phenytoin	3 (3.61)	4 (4.65)
Phenobarbital + Phenytoin	2 (2.40)	5 (5.81)
Carbamazepine	43 (51.8)	18 (20.93)
Others	21 (25.3)	12 (13.95)

* (Anti-epileptic drugs), Figures in the parentheses are in percentage

Sedation was a problem in three children with phenobarbital. Reduction in dose to 3 mg/kg solved the problem. Hyperactivity was observed in six and irritability in two necessitating switch over to phenytoin or carbamazepine. The side-effects of phenobarbital could be easily recognized and reversed promptly after carbamazepine was used. Phenytoin was used in forty-two children alone and in combination, gum hypertrophy and ataxia were observed in two children each (1.8%).

Of the seventy-two patients with seizure hypocalcaemia (less than 9 mg%) was documented in forty-four (61%), fifty-seven (79%) children had raised alkaline phosphatase levels (more than 270 IU).

## Discussion

Cultural attitudes, a lack of prioritization, poor health system infrastructure and inadequate supplies of antiepileptic drugs are responsible for the unacceptable treatment gap. Compared to Phenobarbital, phenytoin, carbamazepine and valproate are 1.6, 4.7 and 5.3 times as expensive respectively.[9] At our center, approximate cost of an AED, for a 20 kg subject per annum is as follows: phenobarbital (60 mg) Rs.126 ($3), phenytoin (100 mg) Rs.83.40 ($2), carbamazepine (200mg) Rs.604.80 ($15), sodium valproate (200mg) Rs1137.60 ($30). However, the status of phenobarbital as an AED is not just because of cost consideration but also owing to its efficacy. Phenobarbital is a broad spectrum anti-convulsant with undisputed efficacy. With Phenobarbital seizure control for more than 5 months was noticed in 81% of patients with associated life changing improvements in social functioning.[10] A study in rural India has shown that its efficacy is comparable with phenytoin[11] and a study in Ecuador and Kenya have shown that there was no significant difference between phenobarbital and carbamazepine in either efficacy or safety.[12–14] Concerns have been expressed about its side-effects. However, evidence for a negative impact on cognition and behaviour is less compelling than generally thought. These side-effects are perhaps over-stated and may not apply to dosages at the lower end of the effective range.[15] A study from Bangladesh has shown that phenobarbital is not associated with excess behavioural side effects when compared with carbamazepine.[16] The strength and generalisability of evidence of serious behavioural and cognitive effects of phenobarbital in recurrent childhood afebrile seizures is controversial, largely because of methodological problems of published trials.[11] The virtues of phenobarbital are no longer promoted. Lack of commercial interest or commercial neglect has allowed this drug to fade away. However, it is still relevant AED in resource limited setting where the choice is not between phenobarbital and new medicament but between phenobarbital and no treatment at all. Drop out rate, although declined from 44.7% to 19.5%, is still a matter of concern. Ineffective therapy may not be the only reason. Travel time and travel cost to collect monthly quota of AED may also contributing to it. Easy availability at nearby centers may further reduce drop out rates.

Children receiving phenobarbital and phenytoin are vulnerable to vitamin D deficiency and from time-to-time, children with epilepsy especially those with neurodevelopmental handicap, are admitted with gross rickets. To study the extent of the problem, serum calcium, phosphorus and alkaline phosphatase were estimated as a proxy for hypovitaminosis D in 72 consecutively enrolled children. Forty four (61%) children had hypocalcemia (less than 9 mg/dl) and fifty seven (79 %) children had raised alkaline phosphatase levels (more than 270 IU/dl). Vitamin D level in a child with refractory seizures, receiving three AED, (Lamotrigine, carbamazepine and clobazam) was 6.2ng/ml (normal 15-20 ng/ml). The seizures were promptly controlled with vitamin D and phenytoin.

Eight cases presented with breakthrough convulsion after a seizure-free-period of five to eighteen months. They received only injection vitamin D. Seven of them remained seizure free, one patient required AED dose enhancement.

Vitamin D deficiency may lead to hypocalcaemia which in turn lowers seizure threshold. AED enhance the risk of vitamin D deficiency. Extent of this problem has been well recognized.[17] Therefore, our protocol includes periodic administration of vitamin D. Although role of vitamin D deficiency is well recognized, only 7% of adults' neurologists and 9% of childrens' neurologists prescribe calcium and vitamin D to their patients who have seizures.[18]

To conclude, phenobarbital with or without phenytoin can effectively control seizures in children. It is an inexpensive option. Periodic administration of vitamin D appears to contribute to seizure control.
